# Median knock-down time as a new method for evaluating insecticide-treated textiles for mosquito control

**DOI:** 10.1186/1475-2875-7-114

**Published:** 2008-06-27

**Authors:** Ole Skovmand, Julien Bonnet, Olivier Pigeon, Vincent Corbel

**Affiliations:** 1 Intelligent Insect Control, 118 Chemin des Alouettes, 34170 Castelnau, France; 2 Laboratoire de Lutte contre les Insectes Nuisibles, IRD, 911 Avenue Agropolis, BP 64 501, 34 394 Montpellier Cedex 5, France; 3 Walloon Agricultural Research Centre (CRA-W), Pesticides Research Department, Rue du Bordia, 11, B-5030 Gembloux, Belgium

## Abstract

**Background:**

Insecticide treated bed nets are major tools for the Roll Back Malaria campaign. There are two types of Long-Lasting Insecticide-treated Nets (LNs) on the market: coated nets and insecticide-incorporated nets. Nets provided to this market need a recommendation from the World Health Organization to be purchased by donors and NGOs. During laboratory study (phase I), the first step consists in evaluating the wash resistance of a new LN product. When insecticide-incorporated nets are washed, it takes time to regenerate the insecticidal activity, i.e. insecticide must migrate to the net surface to be accessible to mosquitoes. The interval of time required for regeneration must be carefully determined to ensure the accuracy of further results. WHOPES procedures currently recommend the determination of the regeneration time by using mortality data. However, as mortality cannot exceed 100%, a LN that regenerates a surface concentration exceeding the dosage for 100% mortality, will have its regeneration time underestimated.

**Methods:**

The Median Knock Down Time (MKDT) was determined as function of insecticide dosage on an inert surface, glass, and on polyester nettings using an acetone solution or a simple emulsion. Dosage response was also established for mortality data. The same method was then applied to a commercially polyethylene netting, currently under WHOPES evaluation, to determine the dynamics of regeneration as function of repeated washings. The deltamethrin content of these nets was estimated by Capillary Gas Chromatography (GC-ECD).

**Results:**

MKDT was a linear function of log insecticide dosage on glass as on nettings. Mortality data were either 0 or 100% for most concentrations except for a narrow range. MKDT was log linear function of total deltamethrin content in a commercial polyethylene net exposed to washings. The regeneration time of this net increased with the number of washes and MKDT became higher. A new, easy and rapid method to determine MKDT is suggested.

**Discussion:**

The MKDT is linearly correlated to log dosage on a given substrate and shows no saturation as mortality data do. It is suited to determine regeneration time of a product that is exposed to a stress, like washing or heating, where the process impacts on the bio-availability of the insecticide. Mortality data are useful for measuring product efficacy, whereas MKDT are better to measure dynamics of surface concentration like regeneration after a stressing process. Change in MKDT can be used to illustrate the loss of insecticide due to washing, but the slope of the curve is product and surface-dependent.

## Background

Long-Lasting Insecticide-treated Nets (LNs) and tarpaulins have been developed for controlling malaria vectors. Among these are products where the insecticide is slowly released from a matrix of a polymer like polyethylene or polypropylene. This technology is basically different from that of the coating technologies. In the latter, all insecticide is present in a coating at the surface of the material, whereas in the first, most of the insecticide is inside the matrix at any time. Upon removal by washing, rubbing or UV destruction, new insecticide must migrate to the surface of the net to regain activity, *i.e*. regeneration. After abrupt removal of insecticide (due to washing) the regeneration time becomes the parameter that determines how fast the tool again becomes effective for mosquito control.

Nets and other tools that target vector control and especially the currently large, donor financed market must pass a series of tests organized by WHOPES (WHO Pesticide Evaluation Scheme) to have a chance to be sold to these international organizations. The WHO procedures recognize the two types of impregnation and describe a protocol that divides these products into regenerating and non-regenerating products, the latter meaning regeneration in less than a day [1]. The protocol describes that a net is washed thoroughly to remove most or all insecticide from the surface. The net is then exposed to bioassay on succeeding days to determine the regeneration time. The method determines the percentage of Knock-Down after 1 hr (KD_60_) and the percentage of mortality after 24 hr. The time required (in days) to reach a stable mortality level is the period required for regeneration of the net [1]. Thereafter, nets are washed or bioassayed at intervals of time determined by this regeneration time to follow the insecticide exhaustion process. A net is regarded as exhausted if mortality drops below 80% and/or KD60 below 95%. Since percentages can never be more than 100%, the method cannot follow the regeneration process happening when 100% mortality is reached. The time for the surface concentration to become stable may be much longer. When this potentially too short interval is used to determine the regeneration time and, therefore, wash intervals, the net may fail in the assay after few washes since not enough time is given for regeneration before the bioassay. On the contrary, it may resist more washes since the amount removed per wash is too low and the regeneration process is interrupted by the next wash.

The median knock-down time (MKDT) does not have the disadvantage of reaching a maximum level. It is expected to be directly correlated to the insecticide concentration on the surface, at least for fast acting insecticides such as pyrethroids [2]. The MKDT method was first developed by Curtis *et al *[3] to obtain a bioassay with a fast data generating because of problems with holding mosquitoes. It was later described in two WHO meeting reports from 1996 and 1998 [4,2] as a better method for measuring loss of insecticide from dipped bed nets, but the method is not included in the current WHOPES procedures for bed net evaluations [1].

The purpose of this study is to examine whether MKDT is dependent on insecticide dosage on various treated materials, and if this method can be used to determine with more accuracy the regeneration time of LNs. As part of this study, a new test method is proposed where the contact to the treated material is forced, and where the mosquitoes rest only on the treated surface and are relatively easy to observe. The increase in MKDT with the amount of insecticide washed out was followed by chemical analysis. This method compared the initial concentration of insecticide in the net with that left after one to 20 washes.

## Methods

### Study design

Regeneration time is defined by WHO [1] as the time passing for the insecticide to stabilize at the surface after removal of the initial surface cover of insecticide in three consecutive washes as measured with mortality data. In this article, the regenerating time was determined by measuring the time for the median knock-down time (MKDT) to become stable. Further, the MKDT was determined before net washing and daily after net washing for each round of five washes until 20 washes (the WHO threshold for LN).

### Mosquitoes

*Anopheles gambiae *s.s. Kisumu strain was used. This strain is fully susceptible to insecticides and has been kept at the LIN (Laboratoire de Lutte contre les Insectes Nuisibles, IRD, Montpellier, France) laboratory since 1992. Two- to five-day old, non blood fed females were used for the bioassays.

### Bioassays

**Figure 1:**
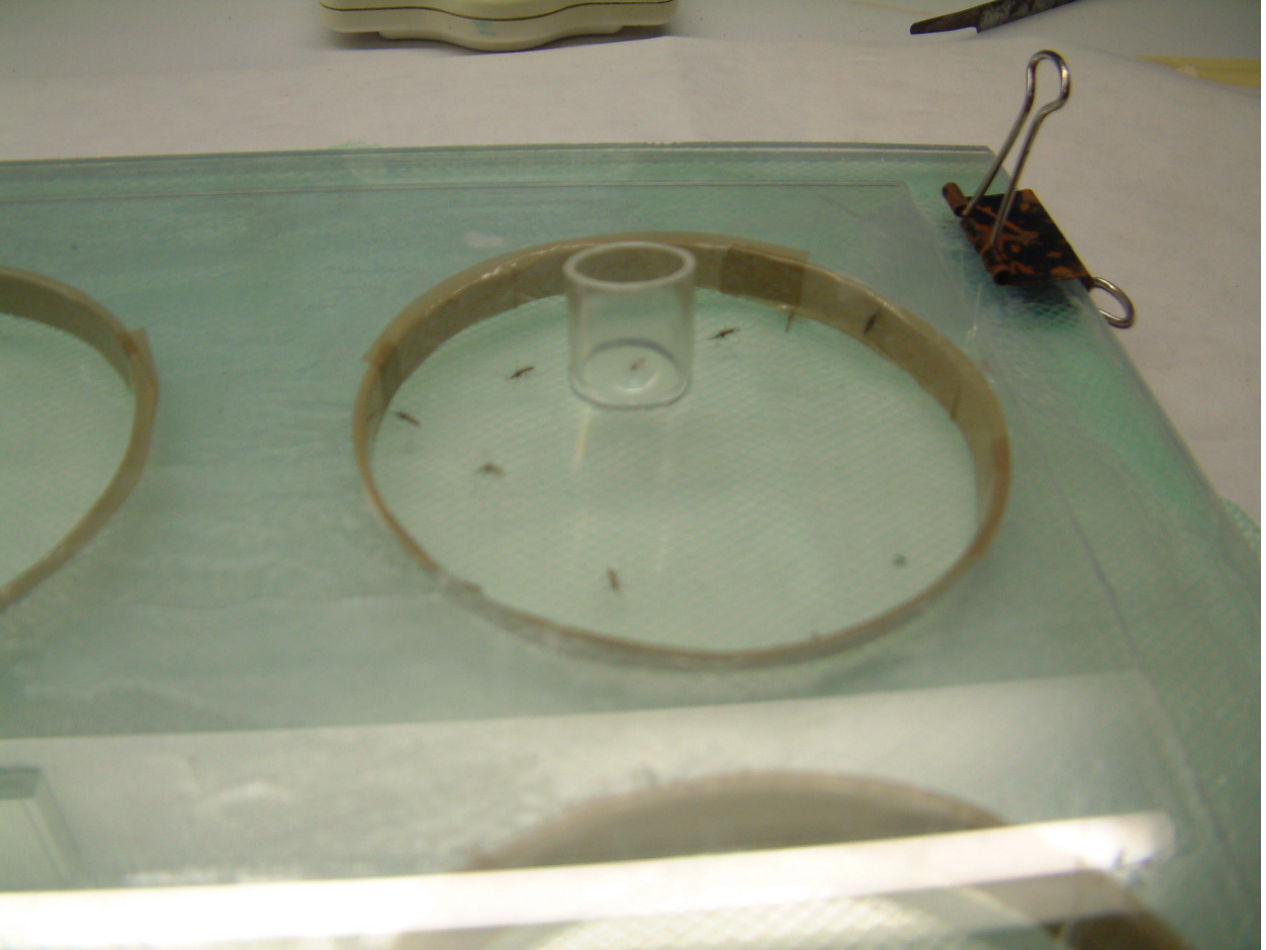
**Exposure device for MKDT**. Mosquitoes are entered under a Plexiglas plate pierced with a hole and a hollow tap to turn the dish and thus easier recapture paralyzed mosquitoes.

Mortality data were obtained from the WHO cone exposure as described in WHO guidelines [1]. In this test, the mosquitoes are introduced into a cone fixed on a net, removed after three minutes and transferred to a cup with sugar water. Knock-down was observed after 60 min (KD_60_) and mortality after 24 hr. All net data presented are based on four independent samples per measurement. In this way, the four samples used for bioassay after e.g. 15 washes were available for chemical analysis. MKDT was determined by introducing mosquitoes, under a glass cover, into a circular chamber (diam: 10 cm, height: 1 cm) cut in a Plexiglas plate. In this method, the net is suspended between the thick Plexiglas plate and a thinner plate with a hole of similar size (Figure 1). The mosquitoes can only walk or stand on the net surface, not fly or rest on other surfaces. Mosquitoes are introduced through a small hole in the glass cover. The hole is closed with a plastic cap during the test. Mosquitoes are relatively easy to observe. The cover glass can be moved freely for fast recovery of the paralyzed individuals. The time for knock-down of each individual mosquito is listed and the median knock down time can be read off from the list (corresponding to the sixth mosquito of a sample of 11 mosquitoes) [2]. Data presented are means of two samples exposed to washing in the same way as the samples used for mortality studies. Data presented from treated glass plates and net swatches are means of three test per concentration and material.

### Insecticide and net materials

Technical deltamethrin from Tagros, India, was used for the impregnations. Serial dilutions of deltamethrin dissolved in acetone were dripped on glass plates or on polyester nets that had been washed once to remove dust and yarn oil. Deltamethrin emulsion was made by dissolving the technical powder in xylene and emulsifying with an oil emulsifier, Polyoxyethylene 10 cetyl ether (Brij 56, Uniqema, Belgium), and hot water. This simple formulation is stable after rapid cooling and used for impregnation of net swatches by dripping the solution on the net.

The polyethylene nets NetProtect (with deltamethrin locked in the matrix) used for the wash resistance study were provided by Intelligent Insect Control, France.

### Washing procedure

The washing procedure followed the protocol described in WHO [1]. In short, 25 × 25 cm net samples are put into 1 L bottles containing 500 ml of soap water (2 g/L). The bottles are introduced in a water bath at 30°C regulated with a thermostat and shaken for 10 min, then transferred to bottles with clean water for rinsing in the same set-up.

### Chemical analysis

The deltamethrin content was determined on unwashed and washed Netprotect samples using the following analytical method. Deltamethrin was extracted from the samples by heating under reflux for 60 minutes with 40 ml xylene. The extract was let to reach ambient temperature, filtered through a Büchner funnel containing a filter paper and a filter aid and quantitatively transferred into a 50 ml volumetric flask. The flask was filled up to volume with xylene. A 10-time dilution was achieved in xylene. The final extract was finally analyzed for determination of deltamethrin by Capillary Gas Chromatography with ^63^Ni Electron Capture Detection (GC-ECD) using the external standard calibration.

### Statistical analysis

MKDT as function of log insecticide concentration was determined for laboratory insecticide treated materials and the slope of the effect curves were compared using Statistix for Windows, 8 (Analytical Software, 2003, Tallassee Fl, US).

## Results

**Figure 2:**
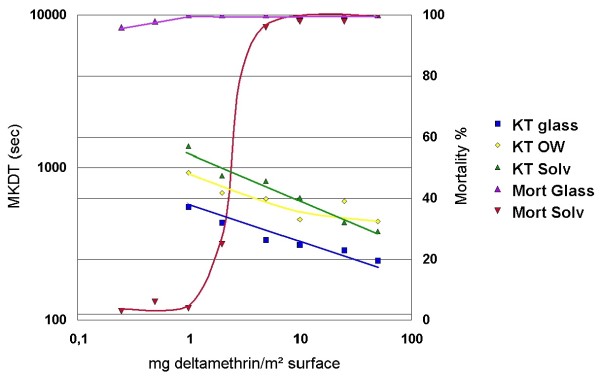
**MKDT and Mortality (%) as functions of insecticide dosage on glass plates and polyester netting impregnated manually**. KT-glass: MKDT from glass plates treated with serial dilution of deltamethrin in solvent (acetone). KT-OW: MKDT from nets treated with serial dilution of deltamethrin in emulsion. KT-Solv: MKDT from nets with serial dilution of deltamethrin in solvent. Mort glass: 24 hr mortality % from glass plates with serial dilution of deltamethrin in solvent. Mort Solv: 24 hr mortality % from nets treated with serial dilution of deltamethrin in solvent. MKDT is log linearly correlated to dosage of insecticide whereas mortality for most concentrations is either near or at 0% or 100%.

The results showed that MKDT declined log linearly with the concentration of deltamethrin in the material treated with serial solutions (Figure 2). Linear correlation analysis of MKDT as function of log doses of deltamethrin (mg/m^2^) had high correlation coefficients (r^2 ^= 0.89, 0.91 and 0.71 for acetone solution on glass plate, on net and emulsion on net, respectively). MKDT values were more dependent on material than on concentrations. For the same concentration, MKDT was much shorter on glass plates than on nets, and again shorter from emulsions dripped on net than from acetone solutions dripped on net (P < 0,001). The slope of the three lines showing log MKDT as function of log doses of deltamethrin were significantly different (*P *= 0.018). The slopes of the two curves describing MKDT of the emulsion on net and of the solution of deltamethrin on the glass plate were not different, whereas both were different from that of deltamethrin acetone solution on net.

Mortality of *An. gambiae *females remained constant at 100% for a wide range of concentrations on glass plates, but dropped sharply within a short interval of concentrations (from 1 to 5 mg/m^2^) for nets treated with an acetone solution of deltamethrin dripped on net (Figure 2).

**Figure 3:**
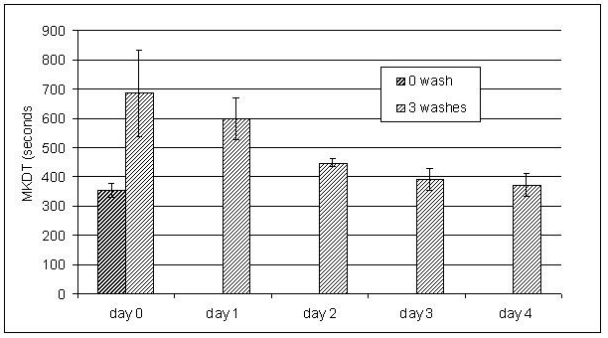
**MKDT on an unwashed and triple washed net from NetProtect on succeeding days showing regeneration**. Three net samples were washed three times on a day to measure regeneration time. MKDT was measured before these washes and daily after. Regeneration time was determined from MKDT that is directly dependent on surface concentration.

**Figure 4:**
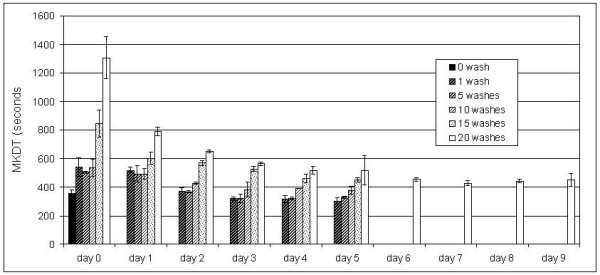
**Dynamics of net regeneration measured by MKDT of netting from NetProtect washed from 0 to 20 times**. Two net samples were tested before and after washing with the wash interval determined currently. After the first wash and tested on the same day (day 0), the MKDT increased and then declined to a stable level in 2–3 days. Samples were then washed 5 times with 3 days interval and then again bioassayed daily for 5 days etc. The MKDT determined immediately after washing increased with the number of washes passed. The time before a steady level was reached became longer and longer.

The polyethylene net NetProtect^® ^initially showed a MKDT of 380 sec and a regeneration time of 2–3 days after three consecutive washes in a day (Figure 3), as judged graphically. Variance analysis shows that only MKDT mean values of day 0 and day 1 after washing were significantly different from the initial value. If the gradual decline in MKDT is described as a log function of time, this log linear decline reached the pre-wash level after three days. The test of three samples showed very little variation between samples. The stable MKDT value was the same after five washes with a three-day interval as the initial value, but then increased with further washing (Figure 4). Further, with increasing number of washes, more and more time was needed to reach a stable level of MKDT (for at least five days after 20 washes).

**Figure 5:**
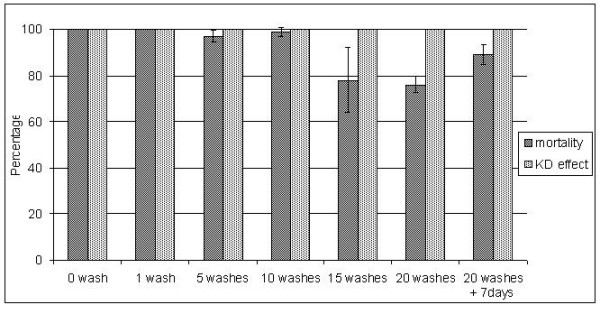
**Mortality at 24 h and KD_60 _of nets from NetProtect after serial washes of 5 till 20 using WHO cone tests**. The Mortality starts to decline below 100% after 15 washes and is below 80% after 20 washes, though KD_60 _is still above 95%. The bioassay after 20 washes was repeated one week later (20+7 days) which showed that after the original regeneration time had passed, the net had not fully recovered. At full recovery, the net still killed more than 80% of the mosquitoes.

After three consecutive washes, mortality took three days to reach a stable level of 100% and remained at 100% until day 5. The succeeding rounds of five washes and a bioassay showed that mortality was close to 100% after the regeneration time until 15 washes and then started to decline (Figure 5). When samples were left an extra week after 20 washes, efficacy was recovered and mortality became around 90%, above the threshold of 80% mortality set by WHO for a net to be effective [1]. Percentage knock-down at 1 hr remained above 90% during the whole test.

**Figure 6:**
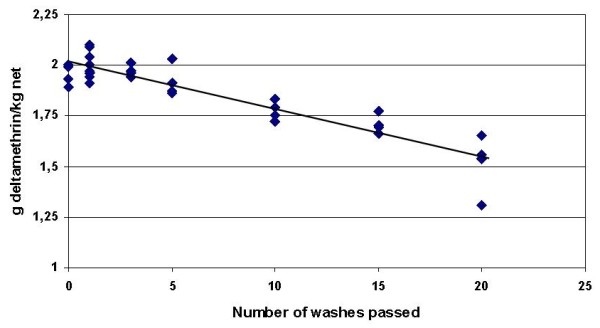
**Decline in total deltamethrin concentration (g/kg net) as function of number of washes passed**. The average wash off rate was 1,23% (retention index of 98,8) and after 20 washes, 77% of the insecticide remained.

Chemical analyses were used to determine the total concentration of deltamethrin in the polyethylene samples before wash, and after 1, 5, 10, 15 and 20 washes. The average wash off rate was calculated from the log linear decline in total concentration of insecticide in the net and determined to be 1.23% (Figure 6). The decline after 20 washes was 23%.

Chemical analyses were carried out on samples drawn from the pool of net samples washed and used in bioassays. These analyses were compared to MKDT values after regeneration, meaning when these values were stable. A log linear relation was then found between MKDT and dosage deltamethrin/m^2^.

## Discussion

New types of bed nets are being made available in the current, worldwide campaign against malaria. Many of these nets are or have been evaluated in the WHOPES in a procedure combining initial laboratory tests followed by semi field tests and evaluation of field tests programmed in co-operation with WHO or accepted as supplementary data.

Whereas the new WHO test protocol [1] uses 24-hour mortality and KD effect only, this study shows that additional information can be obtained by adding median time measure for knock down, MKDT. This complementary method is useful for measuring bio-availability of insecticide on the surface of a net and regeneration time. It has no saturation point and can measure the time for stable surface concentration, even when mortality has gone into saturation at 100% long before. Accordingly, it is a suitable technique to determine regeneration time. The method was originally suggested as a better method than mortality data for evaluating loss of insecticides from dipped nets because it does not saturate at 100% [2], but is not part of the current WHOPES protocol for bed net evaluations [1].

Acetone solutions dripped on glass or polyester net leaves only the deltamethrin on the substrate upon drying. Yet, there is a big difference between the effects of deltamethrin on net to that of deltamethrin on glass plate, the latter being much more effective than the former. It is possible that the acetone let the deltamethrin partly enter into the polyester yarn surface. In both cases, there is a dose-relationship between deltamethrin and MKDT. Interestingly, a simple W/O emulsion of deltamethrin from polyester net gave shorter MKDT values than the solvent solution, probably showing that deltamethrin is easier transferred from the net treated with the emulsion. For all three surfaces/formulations, there is a linear correlation between log concentration of insecticide and MKDT, meaning that MKDT for a given formulation and product can be used to indicate the biologically active surface concentration. A log linear correlation was also found between MKDT measured on a commercial polyethylene net and the dosage of deltamethrin in this net (mg/m^2^) determined in chemical analysis.

Contrary to that, concentrations of the range 0.25 to 50 mg deltamethrin/m^2 ^gave 96–100% mortality on glass plates. For deltamethrin acetone solution dripped on net, mortality changed from near 0 to near 100 in a narrow range between 1 and 5 mg deltamethrin/m^2^. Obviously, mortality data can be used for threshold measurements, but will then be very susceptible to small variations in surface concentration or insect susceptibility.

The data also show that for a commercially available polyethylene net incorporated with deltamethrin, regeneration time increased along with exhaustion of the net by washing: the more the net was washed, the longer it took before a stable level of MKDT was reached. Further, the fact that the stable level of MKDT increased with washings indicates that the stabilized surface concentration declined as the yarn was exhausted of insecticide. This indicates that the process involved is a migration of a chemical from a solid material and can be approached with Fick's second law of diffusion: J(t) = D * C, where C is the concentration of insecticide in the matrix, and D is the diffusion constant. The diffusion rate J(t) changes with the concentration in the yarn. The simplified version of Fick's law presented above ignores the surface concentration that is low after wash off, but as this one increases, the diffusion stops. By repeated removal of the surface layer of insecticide by washing the yarn content of insecticide decreases, the migration rate becomes slower and the surface concentration became lower.

This explanation is supported by the chemical analytical data (Figure 6). These show a log linear decline in total deltamethrin as function of the number of washes corresponding to a wash off rate of 1.23% per wash. This is only possible when the regeneration process continued to stabilization where there is a constant ratio between surface concentration and total concentration. The data do not prove if the washing process removes all insecticide on the surface or just a certain percentage. However, the important point is to know how much a soap washing removed. The combination of washing nets and determining the remaining insecticide thus provide a method for evaluating the wash resistance of a net, a parameter that may be used for estimating the field longevity of a net.

The WHO protocol [1] for regenerating nets operates with a constant regeneration interval between washes. This study shows that the regenerating time increased with the number of washes. Since it is very unlikely that people will wash their nets with few days interval, it can be expected that nets normally will have time enough to regenerate before next washing. Accordingly, tracing the development of the regeneration time along the wash resistance study as done here and then applying it along the exhaustion process gives a realistic picture of the wash resistance of the net.

This study introduced a new way of measuring MKDT (Figure 1). MKDT studies are easy to implement, since the results are obtained within minutes compared to the 24 hours of the mortality bioassay, but several test tools used have given different results. The WHO test tube has been used with a treated or non-treated net at the open end of the tube, giving two different results. Nets have been wrinkled in one [2-4] or two [5] layers around a grid ball, the two layers to avoid that the mosquitoes can escape. However, this also means operating with two different dosages, since mosquitoes now may touch nets in two layers. Yates *et al *[6] also report very little variation in median MKDT in repeated tests, whereas the inter-sample variation found in median MKDT by Graham *et al *[7] from single nets may be due to the heterogeneity of the impregnation of the tested nets. Under more controlled situations, it has been discussed whether the differences in results between laboratories are due to the mosquito's ability to avoid contact with the treated surface in some bioassays (by flying around or sitting on other surfaces than the treated net) [5]. Also, difficulties of seeing the mosquitoes clear enough through the double layer of net may impact on results and variability in readings (John Gimnic, pers. comm.).

By using a transparent glass plate covering a 10 cm round hole in a 10 mm thick Plexiglas plate, these inconveniences are solved. Mosquitoes are easy to observe and the height of the hole allow them to walk around on the net, but not to fly. Therefore, they are in forced contact with the net sample all the time. The hole in the glass lid makes it easy to introduce and remove mosquitoes by moving the plate around above the net sample (Figure 1).

## Conclusion

The present article presents a new way to measure regeneration time. This method is recommended especially for following changes in surface concentrations of insecticide treated materials. Linearity of the method is documented for coated nets as for a commercial insecticide incorporated net. For LNs, the method provides a complementary and reliable way in determining regeneration time after washing.

## Authors' contributions

OS designed the study, wrote most of the text and made the statistical analysis, JB carried out the bioassays and developed the new bioassay method with VC who co-authored the manuscript, OP carried out the chemical assays and described the analytical method. All authors read and approved the final manuscript.
